# Genetic diversity of *Alternaria* species associated with black point in wheat grains

**DOI:** 10.7717/peerj.9097

**Published:** 2020-05-05

**Authors:** Ainur Turzhanova, Oxana N. Khapilina, Asem Tumenbayeva, Vladislav Shevtsov, Olesya Raiser, Ruslan Kalendar

**Affiliations:** 1 National Center for Biotechnology, Nur-Sultan, Kazakhstan; 2 Department of Agricultural Sciences, University of Helsinki, Helsinki, Uusimaa, Finland

**Keywords:** Alternaria, Fungi, Molecular marker, Genetic diversity, Retrotransposon

## Abstract

The genus *Alternaria* is a widely distributed major plant pathogen that can act as a saprophyte in plant debris. Fungi of this genus frequently infect cereal crops and cause such diseases as black point and wheat leaf blight, which decrease the yield and quality of cereal products. A total of 25 *Alternaria* sp. isolates were collected from germ grains of various wheat cultivars from different geographic regions in Kazakhstan. We investigated the genetic relationships of the main *Alternaria* species related to black point disease of wheat in Kazakhstan, using the inter-primer binding site (iPBS) DNA profiling technique. We used 25 retrotransposon-based iPBS primers to identify the differences among and within *Alternaria* species populations, and analyzed the variation using clustering (UPGMA) and statistical approaches (AMOVA). Isolates of *Alternaria* species clustered into two main genetic groups, with species of *A.alternata* and *A.tennuissima* forming one cluster, and isolates *of A. infectoria* forming another. The genetic diversity found using retrotransposon profiles was strongly correlated with geographic data. Overall, the iPBS fingerprinting technique is highly informative and useful for the evaluation of genetic diversity and relationships of *Alternaria* species.

## Introduction

Kazakhstan is an important bread wheat exporter due to the exceptional grain quality and high protein content of wheat crops. Spring wheat is the main export crop in Kazakhstan, grown on approximately 14.3 million ha (FAO, 2013), most of which is concentrated in North Kazakhstan. In this region, climatic conditions favor the development of pathogenic microorganisms in wheat crops, reducing the productivity and quality of grain (https://stat.gov.kz/) (Fehér et al., 2017).

The genus *Alternaria* is widely distributed, and can act as both a saprophyte in plant debris and a plant pathogen (Lawrence et al., 2013). Fungi of the genus *Alternaria* commonly infect cereal crops and cause diseases such as black point and wheat leaf blight, which decrease the yield and quality of cereal products (Woudenberg et al., 2015). Specifically, deterioration of cereal products is caused by mycotoxins produced by *Alternaria* fungi, which can have carcinogenic and allergic effects (Pinto & Patriarca, 2017; Somma et al., 2019; Tralamazza et al., 2018; Wenderoth et al., 2019). Successful breeding work on resistance to fungal diseases requires knowledge of their genetic variability in each ecological region (Xu, 2016). However, precise taxonomic identification poses a substantial challenge, especially for the *Alternaria* genus, which exhibits significant variability in its cultural and morphological characteristics (Shamim et al., 2017). Nevertheless, this remains an important issue to address, as *Alternaria* spp. were found to predominate the mycobiota in wheat from North Kazakhstan’s main wheat-producing area (Gannibal, Klemsdal & Levitin, 2007).

A number of different techniques are currently available to identify genetic differences between organisms and perform comparative analyses based on genomic DNA. One of the most common methods to study the genetic diversity of *Alternaria* is polymorphism analysis of internal transcribed (ITS) and intergenic spacer (IGS) regions of ribosomal DNA (Andersen et al., 2009; Ozer & Bayraktar, 2018). Another class of molecular tools to measure genetic diversity is molecular markers such as RAPD (Random Amplified Polymorphic DNA) (Williams et al., 1990), AFLP (Amplified Fragment Length Polymorphism) (Vos et al., 1995), ISSR (Inter-Simple Sequence Repeat) (Sivolap, Kalendar & Chebotar, 1994; Zietkiewicz, Rafalski & Labuda, 1994), or SSR (Simple Sequence Repeat). Molecular markers have become enormously important because they enable the quantification of genetic diversity, measure inbreeding, characterize new species, taxonomy, and evolutionary origin. However, each type of marker comes with disadvantages including time/labor requirements, cost, speed, effectivity, genome coverage, and degree of polymorphism detection. For example, RAPD is an inexpensive and time-effective technique used to analyze genomic polymorphism among related organisms (Williams et al., 1990). However, this method is sensitive when the PCR conditions change due to mismatches between primer and template, and mismatches can lead to inefficient amplification of targeted regions of DNA template. The ISSR technique is an extended version of the RAPD technique, which retains the same positive and sensitive features as RAPD (Zietkiewicz, Rafalski & Labuda, 1994). RAPD and ISSR are the most common DNA-based techniques that provide highly discriminating information with relatively good reproducibility. Similarly, AFLP analysis has the capability to detect various polymorphisms in different genomic regions simultaneously. However, AFLP is a more complex—and hence time-consuming—method. It involves several steps, including two PCR rounds and restriction-ligation with an adaptor. The AFLP protocol is critically dependent on DNA quality, but is capable of revealing numerous polymorphic bands with just a few primer combinations.

In addition to these DNA profiling methods for investigating genetic variation in fungi, using multicopy and genomic abundance of retrotransposons can extend knowledge of phylogenetic relationships and estimate genetic diversity (Hosid et al., 2012; Kalendar, Amenov & Daniyarov, 2019; Kalendar & Schulman, 2014; Kalendar et al., 2017). Retrotransposon-based DNA profiling applications offer a simple, cost-effective (Gribbon et al., 1999; Kalendar et al., 2011; Kalendar & Schulman, 2006) and highly reproducible way to study genetic polymorphisms. These beneficial features are based on the fact that retrotransposons (in particular, Long Terminal Repeat (LTR) retrotransposons) are distributed throughout the genome and are involved in recombination events that occur during meiosis (Belyayev et al., 2010; Hosid et al., 2012; Schulman & Kalendar, 2005; Vicient et al., 2001; Vicient, Kalendar & Schulman, 2005). Transcriptionally active retrotransposons also play an important role in gene regulation and adaptation to ecological stress, because their activity is induced by stressful environmental conditions (Vicient et al., 2001).

According to the concept of “two-speed” genomes, phytopathogenic fungi potentially cause multiple gene rearrangements produced by retrotransposons. As many plant pathogenic fungi have genomes expanded by retrotransposon insertions, the hypothesis that “bigger can be better” was proposed as a mechanism of antagonistic co-evolution with a host (Raffaele & Kamoun, 2012). Ultimately this promotes rapid evolution of pathogenic microorganisms. The “two-speed” genome concept highlights compartmentalization into repeat-dense regions with higher recombination rates, and gene-dense regions that remains fairly conserved over evolutionary time. The “two-speed” genome hypothesis explains the independence of genes encoding essential housekeeping functions in the core genome, while allowing novel genes to evolve in the accessory genome (Dodds, 2010; Dong, Raffaele & Kamoun, 2015). These findings have led to the “two-speed genome” model in which endogenous fungi genomes have a bipartite architecture with gene-sparse, retrotransposon-rich elements that are thought to contribute to the potential to rapidly evolve virulence.

LTR retrotransposon sequences are commonly used to identify the molecular genetic polymorphism within lines and varieties of plant and animal breeds. Specifically, PCR methods based on detection of transposable element insertion site polymorphisms include Inter-retrotransposon amplified polymorphism (IRAP) (Kalendar et al., 1999), REtrotransposon-Microsatellite Amplified Polymorphism (REMAP) and Sequence-Specific Amplification Polymorphism (SSAP) (Waugh et al., 1997). However, DNA profiling applications based on retrotransposons are limited by a paucity of knowledge about nucleotide sequences of LTR retrotransposons in species without a sequenced genome. In particular, phytopathogenic fungi have a small genome, so the development of genetic markers based on retrotransposons is difficult relative to species with a large genome (e.g., green plants and animals; Mandoulakani et al., 2015; Doungous et al., 2015; Ghonaim et al., 2020; Leigh et al., 2003; Li et al., 2020; Teo et al., 2005; Vukich et al., 2009; Vuorinen et al., 2018). However, the Inter-primer Binding Site (iPBS) amplification technique has proved to be a powerful DNA fingerprinting method that does not require information about retrotransposon sequences (Kalendar et al., 2010). Both retroviruses and LTR retrotransposons use cellular transfer RNAs (tRNAs) as primer molecular to guide the reverse transcription of retrotransposons during their replication cycles. Primer tRNA is selectively packaged into the virion, where it is placed onto the primer binding site (PBS) of the viral RNA genome and the reverse transcriptase (RT)-catalyzed synthesis of minus-strand complementary DNA (cDNA). These LTR retrotransposons and all retroviruses contain a tRNA-conservative PBS, usually for methionine initiator tRNA (tRNAi^Met^).

In the case of retrotransposons, the PBS is either complementary to the 3′ end or to an internal region of the primer tRNA. The iPBS amplification method is based on the virtually universal presence of a tRNA complement as a PBS in LTR retrotransposons that utilize conserved PBS sequences as PCR primers for detection of polymorphism between different individuals, as well as polymorphism within transcription profiles (Monden, Yamaguchi & Tahara, 2014). This method can also be applied for quick cloning of unknown LTR segments from genomic DNA, and for species identification based on information about LTR retrotransposons. The effective iPBS method has been applied to a wide range of studies on plants and animals. The fact that most retrotransposons are nested, inverted, and truncated allows them to be easily amplified from nearly every organism using inverted PBS primers. Moreover, this method can be used as a universal and high-efficiency tool for direct detection of DNA polymorphism (Doungous et al., 2020; Milovanov et al., 2019). Primers that were developed for amplification of the conserved PBS regions showed their effectiveness in the cloning of LTR retrotransposons (Kalendar et al., 2010), including non-autonomous elements that did not contain protein-coding regions such as TRIM (Terminal Repeat Retrotransposons In Miniature) (Kalendar et al., 2008) and LARD (Large Retrotransposon Derivatives) (Kalendar et al., 2004).

Thus far, the application of the iPBS method for investigating the genetic diversity of fungal pathogens has been very limited (Borna et al., 2016; Ozer & Bayraktar, 2018; Özer, Bayraktar & Baloch, 2016; Šķipars et al., 2018; Wu et al., 2019). To date, retrotransposon sequences have been used to study the genetic diversity of only a few *Alternaria* species isolated from wheat seeds. However, the black point disease complex from wheat grains has numerous *Alternaria* species. No genetic diversity studies have been conducted on the most common species, including *A. alternata, A. tenuissima, A. arborescens,* and *A. infectoria,* using the transposable element insertion site polymorphism amplification method. Here, we investigated the genetic relationships of the main *Alternaria* species related to black point disease of wheat in Kazakhstan using the iPBS DNA profiling technique.

## Material and Methods

### Fungal materials and culture conditions

**Figure 1:**
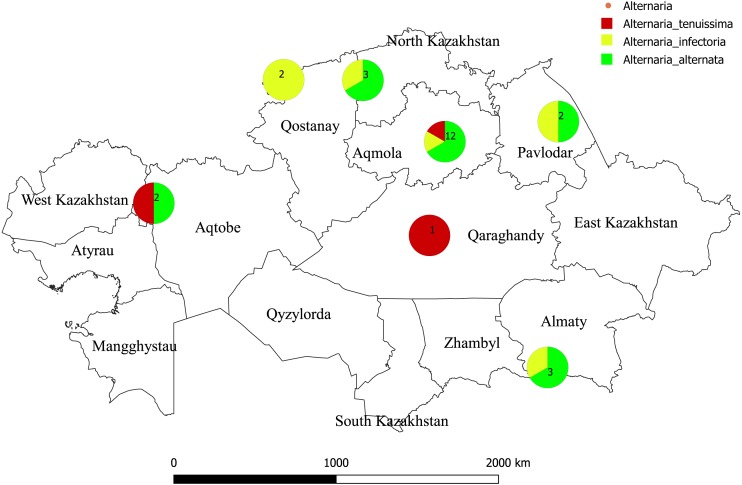
Map of Kazakhstan showing where isolates of Alternaria species were sampled. The color reflects to species of Alternaria. Numbers refers to a total number of species isolated. The geographic location map was drawn using QGIS 3.10.0-GRASS (QGIS Geographic Information System. Open Source Geospatial Foundation) (https://www.qgis.org/en/site/).

In total, 25 single-spore isolates of *Alternaria* sp. were collected from wheat grains of various wheat cultivars from different geographic regions in Kazakhstan (Table 1; Fig. 1). Two hundred seeds were arbitrarily selected from each wheat cultivars. The grains were surface-sterilized by shaking in 10% commercial bleach “Domestos” for 10 min and rinsed three times in sterile water for 1 min each time. Grains were then plated on Petri dishes of potato carrot agar and incubated for 7 days at 25 °C in the light. All isolates were identified based on morphological observation and sequencing of the ITS region. Morphological identification of *Alternaria* species was carried out according to Lawrence et al. (2013) and Lawrence, Rotondo & Gannibal (2015).

**Table 1:** Isolates of *Alternaria* sp. used in this study.

**Species**	**Sources in Kazakhstan**	**ID of isolate**
*Alternaria tenuissima*	Akmola region	2018009
		2018124
	Aktobe region	2018069
	Karaganda region	2018075
*Alternaria infectoria*	Akmola region	2018062
		2018067
	Kostanay region	2018083
		2018128
	Almaty region	2018061
	Pavlodar region	2018041
	North Kazakhstan region	2018056
*Alternaria alternata*	Akmola region	2018013
		2018037
		2018085
		2018123
		2018130
		2018131
		2018133
		2018134
	Almaty region	2018088
		2018122
	North Kazakhstan	2018137
		2018139
	Aktobe region	2018132
	Pavlodar region	2018136

### DNA extraction

*Alternaria* species isolates were grown in Petri dishes containing Chapek media without agar in darkness at 25 °C for one week. Mycelium was scraped with a sterile scalpel and collected into 2-ml tubes. Genomic DNA was extracted from fungal mycelia (50 mg) using modified CTAB extraction buffer (2%, 2M NaCl, 10 mM Na_3_EDTA, 100 mM HEPES, 5.3) with RNAse A treatment (http://primerdigital.com/dna.html). A detailed protocol for DNA isolation was followed as described in Kalendar et al. (2020). The DNA pellets were dissolved with 1 ×TE buffer (1 mM EDTA, 10 mM Tris-HCl, pH 8.0). The DNA quality was checked spectrophotometrically with a Nanodrop apparatus (Thermo Fisher Scientific Inc., Waltham, MA, USA) and also checked by 1% agarose gel, run at 90 V for 20 min.

### PCR protocol for inter-primer binding sites

The genetic variability of *Alternaria* sp. isolates was analyzed by using 25 PBS primers designed by Kalendar et al. (2010). PCR reactions were performed in a 25 µl reaction mixture. Each reaction mixture contained 25 ng of template DNA, 1×Phire^®^ Hot Start II PCR buffer with 1.5 mM MgCl_2_, 1 µM primer, 0.2 mM each dNTP, and 0.2 µl Phire^®^ Hot Start II DNA polymerase (Thermo Fisher Scientific Inc.). PCR amplification was carried out in a Bio-Rad Thermal Cycler T100 under the following conditions: initial denaturation step at 98 °C for 1 min, followed by 30 amplifications at 98 °C for 5 s, at 50−60 °C (depending on primer sequence) for 20 s, and at 72 °C for 60 s, followed by a final extension of 72 °C for 3 min. All PCRs were repeated at least twice for each isolate. All PBS primers were tested to assess the genetic diversity of *Alternaria* isolates using iPBS amplification for DNA profiling. Primers that generated few PCR products were excluded. Primers with a weak profile or that produced mainly monomorphic amplification products were also excluded. PCR products were separated by electrophoresis at 70V for 8 h in 1.2% agarose gel with 1xTBE buffer. A Thermo Scientific (100–10,000 base pairs) GeneRuler DNA Ladder Mix (#SM0332) was used as a standard DNA ladder. The PCR products were visualized with a ChemiDoc-It2 Imaging System (UVP, LLC, Upland, CA, USA; Analytik Jena AG, Jena, Germany) and a PharosFX Plus Imaging System (Bio-Rad Laboratories Inc., Hercules, CA, USA) with a resolution of 50 µm, after staining with ethidium bromide. PBS primers generated in the PCR yielded clearly distinct amplification products, showing considerable variability among the isolates belonging to different *Alternaria* species.

### Data scoring and analysis

Only clear scorable bands were used for studying genetic variability among the isolates of *Alternaria* sp. from wheat grains. Each band of a unique size was assumed to correspond to a unique locus. To construct a binary matrix, reproducible fragments were scored as present (1) or absent (0). GenAlex 6.5 (Peakall & Smouse, 2012) was used to calculate the total number of alleles, Shannon information index (I), genetic differentiation index (PhiPT) among populations, and the number of private alleles per population. Analysis of molecular variance (AMOVA) among and within populations was also calculated with GenAlex 6.5. A dendrogram was constructed using the UPGMA method in MEGA X software (Kumar et al., 2018).

## Results

### PCR amplicon polymorphisms

**Figure 2:**
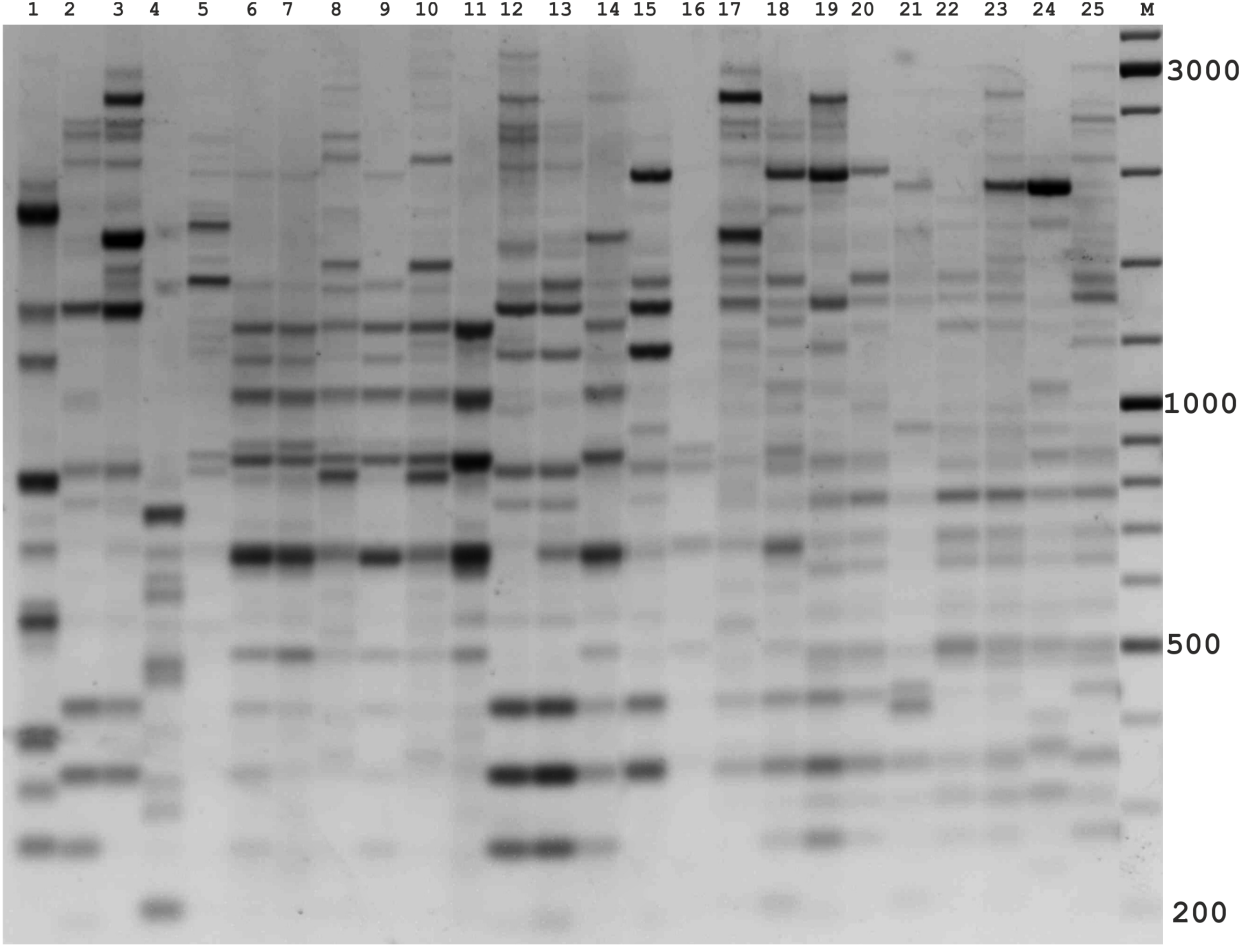
Electrophoretic analysis of PCR product from iPBS marker 2242. Sample order (1–4 *Alternaria tenuissima*, 5-11 *Alternaria infectoria*, 12-25 *Alternaria alternata*) listed in Table 1. M-Thermo Scientific GeneRuler DNA Ladder Mix (100–10,000 bp).

**Figure 3:**
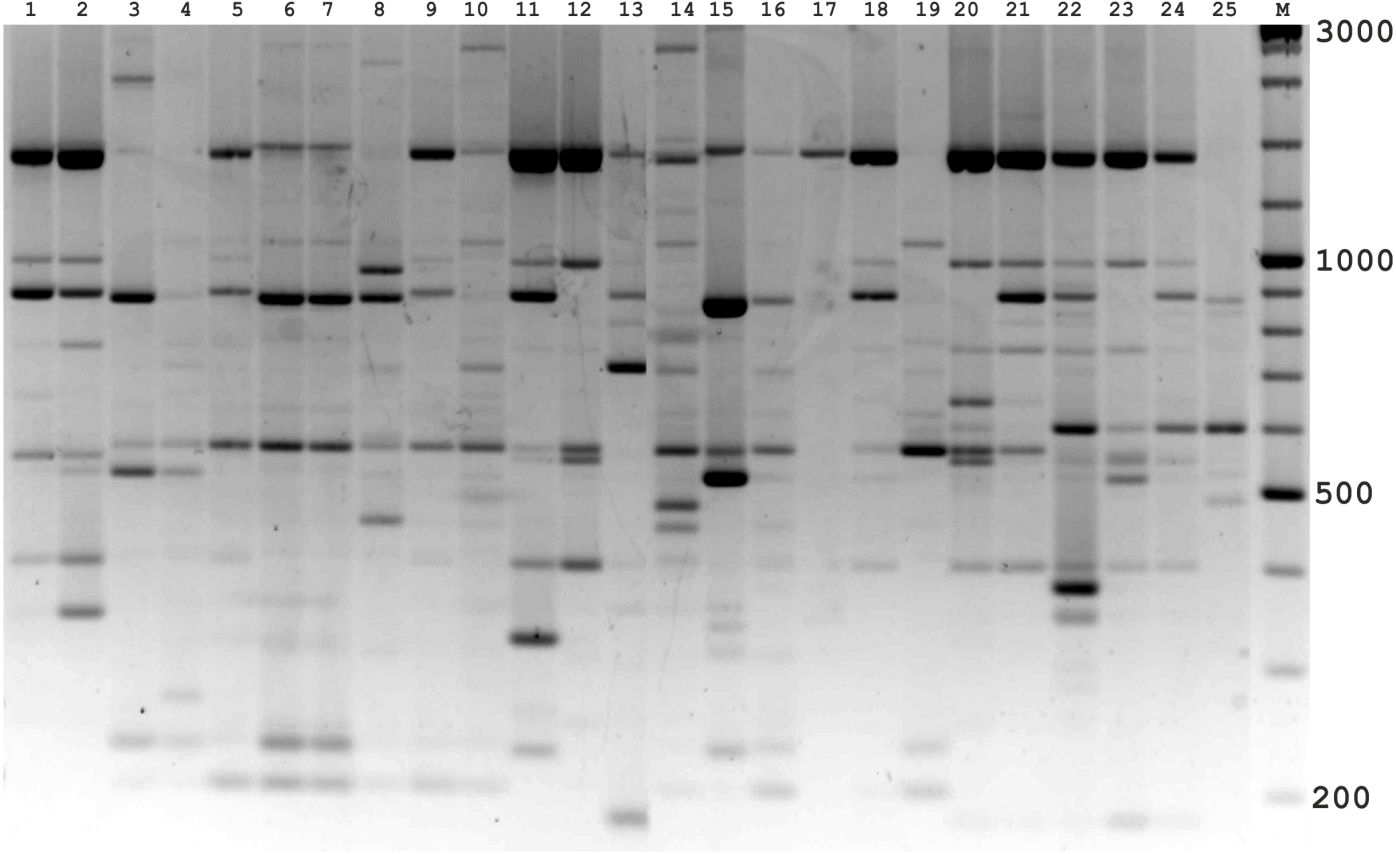
Electrophoretic analysis of PCR product from iPBS marker 2237. Sample order (1-4 *Alternaria tenuissima*, 5-11 *Alternaria infectoria*, 12-25 *Alternaria alternata*) listed in Table 1. M-Thermo Scientific GeneRuler DNA Ladder Mix (100–10,000 bp).

**Figure 4:**
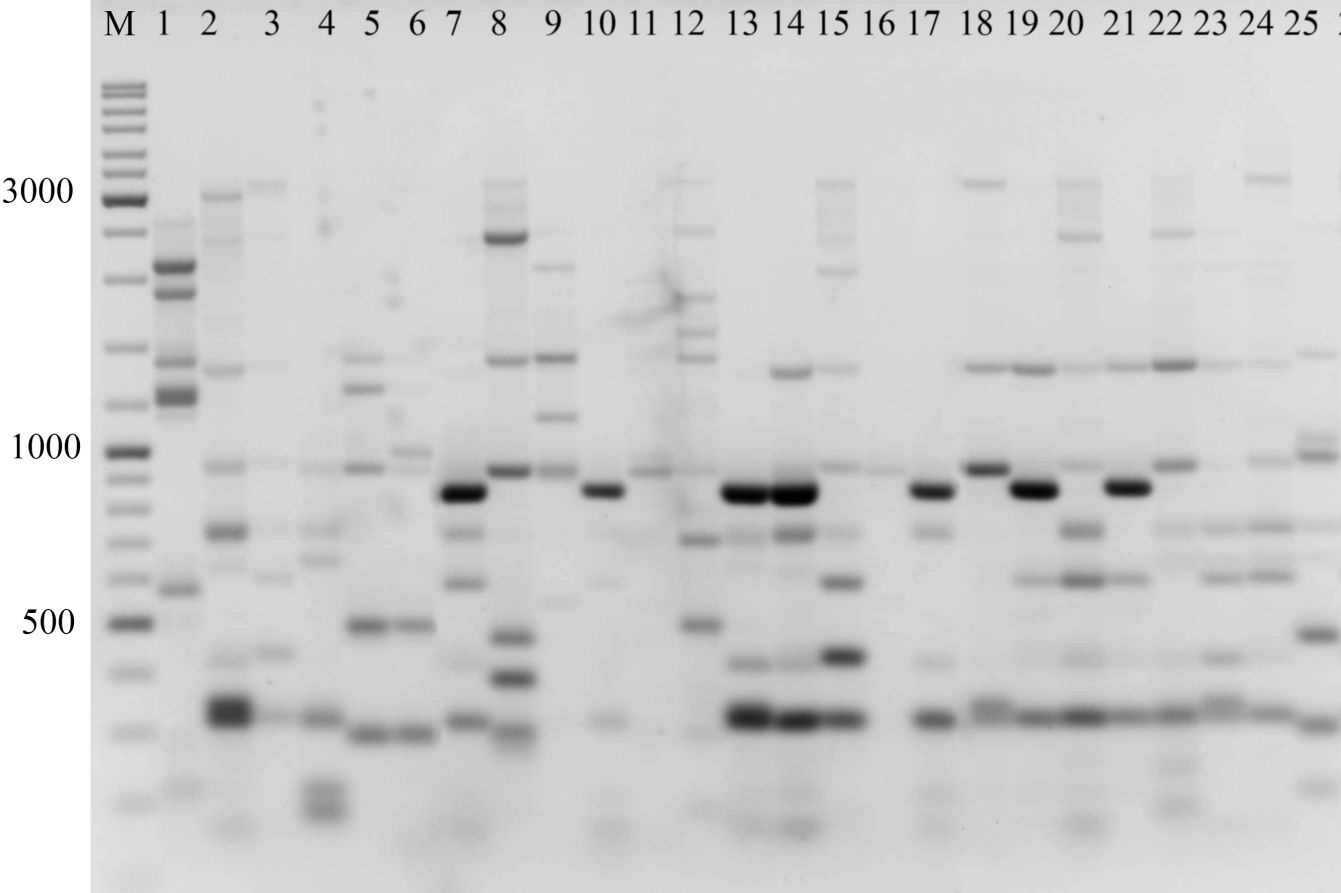
Electrophoretic analysis of PCR product from iPBS marker 2221 Sample order (1-4 *Alternaria tenuissima*, 5-11 *Alternaria infectoria*, 12-25 *Alternaria alternata*) listed in Table 1. M-Thermo Scientific GeneRuler DNA Ladder Mix (100–10,000 bp).

In the preliminary tests, all PBS primers were screened to evaluate their ability to produce clear banding profiles among the isolates. In total, 25 18-mer PBS primers were used (Kalendar et al., 2010). The amplification profile of the PBS primers presented a unique combination of reproducible and scorable bands ranging from 100 to 10,000 bp (Table 2). The iPBS fingerprinting pattern of the fungi genotypes from three primers (2221, 2237, 2242) are shown in Figs. 2–4. The number of amplified bands varied from 15 to 40. On average, each primer generated 20 bands in the profile, with an average of eight that were polymorphic. All PBS primers used for DNA amplification generated a total of 328 scorable reproducible bands (Table 2).

**Table 2:** PBS primers used in the analyses of genetic polymorphism of *Alternaria* sp.

Primer ID	Sequence (5′- 3′)	Tm^∘^ C^a^	TL	PL	PPL (%)	PIC	Range of amplicons (bp)
2242	GCCCCATGGTGGGCGCCA	69.2	19	19	100	0.951	200–3,000
2221	ACCTAGCTCACGATGCCA	58.0	18	18	100	0.941	100–3,000
2237	CCCCTACCTGGCGTGCCA	65.0	16	16	100	0.924	100–3,000
2217	ACTTGGATGTCGATACCA	52.5	5	2	60	0.251	100–2,500
2245	GAGGTGGCTCTTATACCA	53.1	5	2	40	0.258	200–3,000
2253	TCGAGGCTCTAGATACCA	53.4	9	3	33	0.190	200–3,000
2232	AGAGAGGCTCGGATACCA	56.6	6	2	33	0.124	100-2.000
2225	AGCATAGCTTTGATACCA	50.5	10	3	30	0.122	300–3,000
2228	CATTGGCTCTTGATACCA	51.9	7	2	28	0.132	100–3,000
2251	GAACAGGCGATGATACCA	54.3	7	2	28	0.175	100–4.000
2249	AACCGACCTCTGATACCA	54.7	11	3	27	0.168	300–10,000
2220	ACCTGGCTCATGATGCCA	59.0	8	2	25	0.135	300–2,500
2246	ACTAGGCTCTGTATACCA	50.9	9	2	22	0.154	200–3,000
2219	GAACTTATGCCGATACCA	51.5	9	2	22	0.120	100–2,500
2395	TCCCCAGCGGAGTCGCCA	66.0	5	1	20	0.161	100–3,000
2230	TCTAGGCGTCTGATACCA	54.0	15	3	20	0.119	100–10,000
2398	GAACCCTTGCCGATACCA	57.1	16	3	18	0.118	400–2,500
2218	CTCCAGCTCCGATTACCA	56.1	6	1	16	0.115	200–4,000
2222	ACTTGGATGCCGATACCA	55.7	12	2	16	0.118	300–10,000
2226	CGGTGACCTTTGATACCA	54.2	12	2	16	0.114	200–3,000
2255	GCGTGTGCTCTCATACCA	57.1	13	2	15	0.113	100–3,000
2244	GGAAGGCTCTGATTACCA	53.7	20	3	15	0.117	100–3,000
2224	ATCCTGGCAATGGAACCA	56.6	14	2	14	0.117	100–10,000
2243	AGTCAGGCTCTGTTACCA	54.9	7	1	14	0.115	100–3,000
2229	CGACCTGTTCTGATACCA	53.5	9	1	11	0.112	300-2,500

**Notes.**

a T_m_ melting temperature, calculated with 1 µM concentration and without Mg^2+^ (Kalendar et al., 2017a, Kalendar et al., 2017b).

The amplification profiles for three PBS primers (2221, 2237, 2242) were extremely useful compared to the other PBS primers. Among these, primer 2242 showed the highest PIC index value. DNA profiling using PBS primers was highly efficient for isolates belonging to different types of *Alternaria*. The generated amplification products were significantly variable among isolates, both within and among species.

### Genetic diversity among *Alternaria* sp.

In total, 25 PBS primers were used to analyze the polymorphism of 25 *Alternaria* isolates. Several PBS primers showed a high level of polymorphism and were used in further studies to investigate the genetic diversity of other fungal species. Primers with a weak profile or that produced mainly monomorphic amplification products were excluded.

Of the 328 amplified fragments, 228 (69%) were polymorphic (see Table 3 for genetic diversity statistics). The main diversity in the iPBS profile arose from *Alternaria alternata* isolates (160 polymorphic bands out of 198), which was the most abundant species among the isolates (15 out of 25). Notably, the level of detectable polymorphism detected in our study is lower than that reported in similar studies using the iPBS method on both plants (Doungous et al., 2015; Doungous et al., 2020) and other fungi species (Milovanov et al., 2019; Monden, Yamaguchi & Tahara, 2014; Teo et al., 2005; Vukich et al., 2009). The amplified PCR products ranged from 200 to 3000 bp and had on average 10-30 bands per isolate. The percent of polymorphic loci (PPL%) among *Alternaria* sp. were ranked in the following descending order: *Alternaria alternata* (80%), *Alternaria tenuissima* (59%), and *Alternaria infectoria* (47%).

**Table 3:** Genetic diversity of *Alternaria sp* based on iPBS fingerprinting.

**No**	**Species**	**NI**	**NTI**	**PPL (%)**	**PB**	**NPB**
1	*Alternaria alternate*	15	198	80.8%	160	9
2	*Alternaria infectoria*	6	78	47.4%	37	3
3	*Alternaria tenuissima*	4	52	59.6%	31	4
**-**	**Total**	**25**	**328**	**61.0%**	**228**	**16**

**Notes.**

NI number of isolates NTI number of bands per genotype PPL% percentage of polymorphic loci PB number of polymorphic bands NPB number of private bands

Analysis of molecular variance (AMOVA) was used to calculate the number of effective alleles per locus (Ne) based on three PBS primers (2221, 2237, 2242) (Table 4). Ne ranged from 1.189 (*A. infection*) to 1.310 (*A. alternata*). AMOVA revealed that 79% of the total variation was due to differences among isolates within populations, and the variation between populations reflected only 21% of the total variation. These results are also consistent with the low Shannon’s indices (0.198–0.315). The overall Shannon’s index (*I* = 0.266) suggests that more than 20% of the genetic diversity is explained by differences between isolates. Based on these results, we conclude that most of the genetic variation (79%) was distributed among isolates across the regions. It is worth mentioning that the fungal isolates are mostly similar at the genetic level despite long distances between different wheat growing zones in Kazakhstan.

**Table 4:** Analysis of molecular variance (AMOVA) for 25 isolates of *Alternaria* sp based on iPBS fingerprinting.

**Source**	**df**	**SS**	**MS**	**Est. Var.**	**%**	**PhiPT**	**P**
Among Pops	2	38.333	19.167	1.771	21%	0.206	0.001
Within Pops	22	150.467	6.839	6.839	79%		
**Total**	**24**	**188.800**		**8.611**	**100%**		

**Notes.**

Df degree of freedom SS sum of squares MS mean square Est. Var. estimated variance PhiPT genetic differentiation index among populations

The expected variation among groups was 1.771, while within groups it was 6.839, and 8.611 for the total. Hence, the genetic variance was mainly attributed to genetic diversity within groups. The genetic difference (PhiPT) between the three groups was high (0.206). GenAlex 6.5 software (Peakall & Smouse, 2012) was used to analyze iPBS profile data. The number of different alleles found within each population (Ne) and the number of effective alleles per locus were generally higher in the *Alternaria alternata* samples (Table 5). The PIC values ranged from 0.939 to 0.940, and all PBS loci were highly informative (0.5 < PIC < 0.25).

**Table 5:** Summary of *Alternaria* species diversity indices calculated on the basis of iPBS markers.

**Species**	**N**	**Na**	**Ne**	**I**	**He**	**uHe**	**PIC**
*Alternaria tenuissima*	4	1.113	1.304	0.284	0.187	0.214	0.940
*Alternaria infectoria*	6	1.038	1.189	0.198	0.123	0.135	0.940
*Alternaria alternata*	15	1.623	1.310	0.315	0.197	0.204	0.939

**Notes.**

N Number of isolates Na number of alleles Ne number of effective alleles per locus I Shannon’s Information Index He expected heterozygosity uHe unexpected heterozygosity

**Figure 5:**
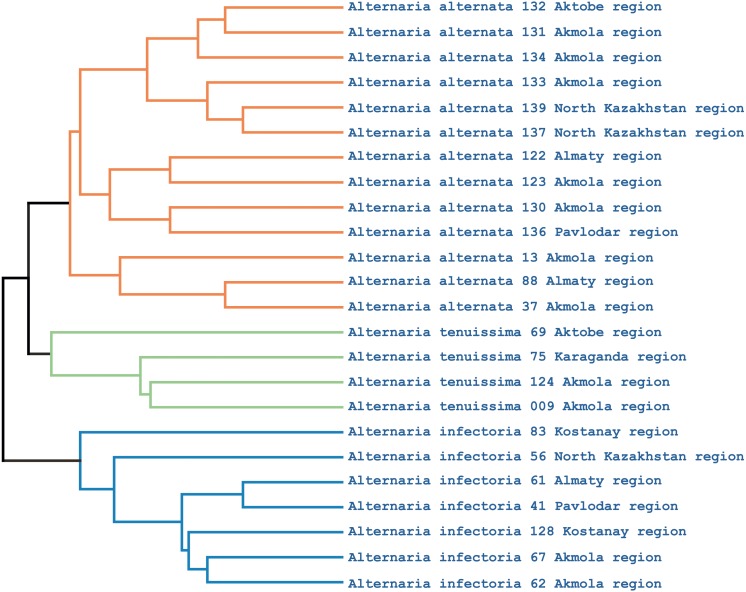
UPGMA dendrogram of 25 *Alternaria* isolates generated from three iPBS primers. Isolates for each species are allocated in separate branches.

Phylogenetic analysis showed that the iPBS markers were effective at grouping the 25 *Alternaria* isolates at the species level. The UPGMA dendrogram grouped all 25 isolates (which represented three populations) into two major clusters (Fig. 5). Among these, 18 and 7 isolates were grouped in clusters 1 and 2, respectively. Although half of the *A. alternata* isolates were collected from the Akmola region, their position in the dendrogram indicates similarity with isolates from other regions. Only two isolates from the Akmola region (137 and 139) were allocated to a separate sub-cluster and had some genetic similarities. Isolates of *A. infectoria* clearly formed a separate cluster. This species is also very different morphologically from *A. alternata* and *A. tenuissima*. Isolates of *A. alternata* and *A. tenuissima* were located in the same sub-cluster, although they are separated from each other.

## Discussion

Resolving the taxonomy of the genus *Alternaria* is a very challenging task because this genus is characterized by significant polymorphisms in morphological and cultural features, as well as biological properties. For example, *A. alternata* and *A. tenuissima* are the most morphologically similar; (Nilsson et al., 2014) also showed that the ITS profiles of these species are 100% identical. Differences between the two species were revealed only at the level of plasma membrane ATPase and calmodulin loci (Lawrence, Rotondo & Gannibal, 2015). Moreover, there is some controversy regarding the harmfulness of *A. alternata* and *A. infectoria* species on wheat plants. This is likely due to the complexity of identifying the species composition of these fungi. Although it is an endophyte, its status as a pathogen is undetermined because it does not synthesize known mycotoxins.

Pathogenic fungi species can infect small grain cereals (wheat, barley, and oat), causing losses by seedling blight, reduced seed germination, or seedling foot and stalk rot. Another potential risk is the presence of fungi toxins; not only do they contaminate cereals, but they could also result in harmful contamination of foods and feedstuffs. Microscopic fungi of the *Alternaria* genus are most often isolated from wheat seeds. These fungi are the dominant component of the grain microbiome in many regions of the world. Considering the significant danger of toxigenic species of *Alternaria*, these fungi have recently received much attention (Patriarca, 2016; Tralamazza et al., 2018).

Identifying the genetic variability in populations within a particular type of *Alternaria* species is important for the development of strategies to counter these fungi, i.e., for breeding programs. Various genetic markers and fingerprinting technologies (RAPD, ISSR, AFLP, and SSR) have been widely used to identify the genetic diversity of fungal populations. Much research has been carried out on other *Alternaria* species (*A. solani, A. brassicicola*) that cause vegetable diseases. However, for species that contaminate wheat, the information is very limited. Methods used to study genetic diversity are technically complicated and costly or have low efficiency. PCR-based DNA profiling technologies based on interspersed repeat sequences such as retrotransposons (Kalendar, Amenov & Daniyarov, 2019; Kalendar et al., 2011) have been intensively employed. In eukaryote genomes, LTR retrotransposons are the major repetitive sequence class and have a high density across the genome. Moreover, stress and adaptation are powerful forces shaping the distribution and accumulation of retrotransposons (Belyayev et al., 2010; Ramallo, Kalendar & Schulman, 2008; Schulman & Kalendar, 2005). Thus, the success and diversity of retrotransposons in a genome are shaped by both the properties intrinsic to the elements and the evolutionary forces acting at the host-species level. Clarification of how these forces act together is paramount to understanding the impact of retrotransposons on organismal biology.

The structure of retrotransposons contains conserved sites that belong to typical retroviruses for all eukaryotes. In this regard, the iPBS method developed by Kalendar et al. (2010) has advantages for applications in the evaluation of genetic diversity, because it allows direct detection of polymorphism regardless of the eukaryotic species. It is particularly beneficial when detecting genetic diversity among fungi isolates; since it can detect polymorphisms in many anonymous loci across the genome simultaneously, it is a highly effective method for studying clonal variability (Doungous et al., 2020; Kalendar et al., 2010; Milovanov et al., 2019). Moreover, since most of the retrotransposons are often mixed with each other, the PCR process amplifies many products because the primers are designed to target conserved regions of retrovirus and LTR retrotransposon primer binding sites. Retrotransposon activity or recombination events lead to novel genomic polymorphisms, which can be detected by this method and used to identify reproductively isolated lines (Mascagni et al., 2017; Sanchez et al., 2017; Underwood, Henderson & Martienssen, 2017).

## Conclusion

In conclusion, this study demonstrated the effectiveness of iPBS amplification for DNA profiling and identification of the endophytic fungi *Alternaria* species in wheat grains. Interestingly, the genetic diversity found here using retrotransposon profiles was strongly correlated with geographic data. One explanation for this observation is that the scored retrotransposon polymorphisms in fungal genomes are related to ecological and environmental stresses. Moreover, retrotransposons in fungal genomes are usually clustered near genes, and thus most likely to be under selection. Finally, permanent changes in retrotransposon content dynamically change fungal genomes; even strains of a single fungal species can display a certain percentage of variability during cultivation in response to different environmental conditions. Abiotic and biotic stresses, including plant interaction, are well known to activate retrotransposons (Belyayev et al., 2019; Kalendar et al., 2000; Ramallo, Kalendar & Schulman, 2008). The iPBS marker analysis allowed us to determine the genetic diversity and population structure of *Alternaria* species isolates and identify various *Alternaria* species. This knowledge may be helpful in understanding host adaptation to this pathogen; knowledge of population genetic structure of a pathogen provides information about its potential to overcome host genetic resistance. iPBS markers could be a useful tool for studying population biology and genetics of this fungus at a global level. The results show rapid LTR retrotransposon evolution in endophytic fungal genomes through integration, losses, and transfers of retrotransposons in almost every species and strain (Giraud et al., 2008). The relationships between the plant host and the endophytic fungal genome can potentially influence the quantity and quality of LTR retrotransposons and the host ecological niche. Hence, the retrotransposon-based DNA profile is highly informative, enabling geographic resolution of *Alternaria* species and giving insight into local factors that may be driving genome adaptation. In addition, DNA profiling based on retrotransposons can be an inexpensive way to establish genetic diversity and to determine the species of fungi.

##  Supplemental Information

10.7717/peerj.9097/supp-1 Supplemental Information 1 A binary matrix

## Additional Information and Declarations

### Data Availability

The following information was supplied regarding data availability: For each locus, data were recorded, using 1 for presence of a band and 0 for absence to build a binary matrix. This data is available as a Supplementary File.

## References

[ref-1] Andersen B, Sorensen JL, Nielsen KF, Gerrits van den Ende B, De Hoog S (2009). A polyphasic approach to the taxonomy of the Alternaria infectoria species-group. Fungal Genetics and Biology.

[ref-2] Belyayev A, Josefiová J, Jandová M, Kalendar R, Krak K, Mandák B (2019). Natural history of a satellite dna family: from the ancestral genome component to species-specific sequences, concerted and non-concerted evolution. International Journal of Molecular Sciences.

[ref-3] Belyayev A, Kalendar R, Brodsky L, Nevo E, Schulman AH, Raskina O (2010). Transposable elements in a marginal plant population: temporal fluctuations provide new insights into genome evolution of wild diploid wheat. Mobile DNA.

[ref-4] Borna F, Luo S, Ahmad NM, Nazeri V, Shokrpour M, Trethowan R (2016). Genetic diversity in populations of the medicinal plant Leonurus cardiaca L. revealed by inter-primer binding site (iPBS) markers. Genetic Resources and Crop Evolution.

[ref-5] Dodds PN (2010). Plant science. Genome evolution in plant pathogens. Science.

[ref-6] Dong S, Raffaele S, Kamoun S (2015). The two-speed genomes of filamentous pathogens: waltz with plants. Current Opinion in Genetics and Development.

[ref-7] Doungous O, Kalendar R, Adiobo A, Schulman AH (2015). Retrotransposon molecular markers resolve cocoyam (Xanthosoma sagittifolium) and taro (Colocasia esculenta) by type and variety. Euphytica.

[ref-8] Doungous O, Kalendar R, Filippova N, Ngane BK (2020). Utility of iPBS retrotransposons markers for molecular characterization of African Gnetum species. Plant Biosystems.

[ref-9] FAO (2013). Perspectivas de cosechas y situación alimentaria. No 1 marzo 2013. http://www.fao.org/3/a-al998s.pdf.%20Consultado%20el%2012/10/14.

[ref-10] Fehér I, Lehota J, Lakner Z, Kende Z, Bálint C, Vinogradov S, Fieldsend A (2017). Kazakhstan’s wheat production potential. The eurasian wheat belt and food security.

[ref-11] Gannibal PB, Klemsdal SS, Levitin MM (2007). AFLP analysis of Russian Alternaria tenuissima populations from wheat kernels and other hosts. European Journal of Plant Pathology.

[ref-12] Ghonaim M, Kalendar R, Barakat H, Elsherif N, Ashry N, Schulman AH (2020). High-throughput retrotransposon-based genetic diversity of maize germplasm assessment and analysis. Molecular Biology Reports.

[ref-13] Giraud T, Refregier G, Le Gac M, De Vienne DM, Hood ME (2008). Speciation in fungi. Fungal Genetics and Biology.

[ref-14] Gribbon B, Pearce S, Kalendar R, Schulman A, Paulin L, Jack P, Kumar A, Flavell A (1999). Phylogeny and transpositional activity of Ty1-copia group retrotransposons in cereal genomes. Molecular and General Genetics.

[ref-15] Hosid E, Brodsky L, Kalendar R, Raskina O, Belyayev A (2012). Diversity of long terminal repeat retrotransposon genome distribution in natural populations of the wild diploid wheat aegilops speltoides. Genetics.

[ref-18] Kalendar RN, Aizharkyn KS, Khapilina ON, Amenov AA, Tagimanova DS (2017a). Plant diversity and transcriptional variability assessed by retrotransposon-based molecular markers. Vavilovskii Zhurnal Genetiki i Selektsii.

[ref-19] Kalendar R, Amenov A, Daniyarov A (2019). Use of retrotransposon-derived genetic markers to analyse genomic variability in plants. Functional Plant Biology.

[ref-20] Kalendar R, Antonius K, Smykal P, Schulman AH (2010). iPBS: a universal method for DNA fingerprinting and retrotransposon isolation. Theoretical and Applied Genetics.

[ref-21] Kalendar R, Flavell AJ, Ellis THN, Sjakste T, Moisy C, Schulman AH (2011). Analysis of plant diversity with retrotransposon-based molecular markers. Heredity.

[ref-22] Kalendar R, Grob T, Regina M, Suoniemi A, Schulman A (1999). IRAP and REMAP: two new retrotransposon-based DNA fingerprinting techniques. Theoretical and Applied Genetics.

[ref-65] Kalendar R, Khassenov B, Ramanculov E, Samuilova O, Ivanov KI (2017b). FastPCR: An in silico tool for fast primer and probe design and advanced sequence analysis. Genomics.

[ref-17] Kalendar R, Raskina O, Belyayev A, Schulman AH (2020). Long tandem arrays of Cassandra retroelements and their role in genome dynamics in plants. International Journal of Molecular Sciences.

[ref-23] Kalendar R, Schulman AH (2006). IRAP and REMAP for retrotransposon-based genotyping and fingerprinting. Nature Protocols.

[ref-24] Kalendar R, Schulman AH (2014). Transposon-Based Tagging: IRAP, REMAP, and iPBS. Methods in Molecular Biology.

[ref-25] Kalendar R, Tanskanen J, Chang W, Antonius K, Sela H, Peleg O, Schulman AH (2008). Cassandra retrotransposons carry independently transcribed 5S RNA. Proceedings of the National Academy of Sciences of the United States of America.

[ref-26] Kalendar R, Tanskanen J, Immonen S, Nevo E, Schulman AH (2000). Genome evolution of wild barley (*Hordeum spontaneum*) by BARE-1 retrotransposon dynamics in response to sharp microclimatic divergence. Proceedings of the National Academy of Sciences of the United States of America.

[ref-27] Kalendar R, Vicient CM, Peleg O, Anamthawat-Jonsson K, Bolshoy A, Schulman AH (2004). Large retrotransposon derivatives: abundant, conserved but nonautonomous retroelements of barley and related genomes. Genetics.

[ref-28] Kumar S, Stecher G, Li M, Knyaz C, Tamura K (2018). MEGA X: molecular evolutionary genetics analysis across computing platforms. Molecular Biology and Evolution.

[ref-29] Lawrence DP, Gannibal PB, Peever TL, Pryor BM (2013). The sections of Alternaria: formalizing species-group concepts. Mycologia.

[ref-30] Lawrence DP, Rotondo F, Gannibal PB (2015). Biodiversity and taxonomy of the pleomorphic genus Alternaria. Mycological Progress.

[ref-31] Leigh F, Kalendar R, Lea V, Lee D, Donini P, Schulman A (2003). Comparison of the utility of barley retrotransposon families for genetic analysis by molecular marker techniques. Molecular Genetics and Genomics.

[ref-32] Li S, Ramakrishnan M, Vinod KK, Kalendar R, Yrjälä K, Zhou M (2020). Development and deployment of high-throughput retrotransposon-based markers reveal genetic diversity and population structure of asian bamboo. Forests.

[ref-33] Abdollahi Mandoulakani B, Yaniv E, Kalendar R, Raats D, Bariana HS, Bihamta MR, Schulman AH (2015). Development of IRAP- and REMAP-derived SCAR markers for marker-assisted selection of the stripe rust resistance gene Yr15 derived from wild emmer wheat. Theoretical and Applied Genetics.

[ref-34] Mascagni F, Giordani T, Ceccarelli M, Cavallini A, Natali L (2017). Genome-wide analysis of LTR-retrotransposon diversity and its impact on the evolution of the genus Helianthus (L.). BMC Genomics.

[ref-35] Milovanov A, Zvyagin A, Daniyarov A, Kalendar R, Troshin L (2019). Genetic analysis of the grapevine genotypes of the Russian Vitis ampelographic collection using iPBS markers. Genetica.

[ref-36] Monden Y, Yamaguchi K, Tahara M (2014). Application of iPBS in high-throughput sequencing for the development of retrotransposon-based molecular markers. Current Plant Biology.

[ref-37] Nilsson RH, Hyde KD, Pawłowska J, Ryberg M, Tedersoo L, Aas AB, Alias SA, Alves A, Anderson CL, Antonelli A, Arnold AE, Bahnmann B, Bahram M, Bengtsson-Palme J, Berlin A, Branco S, Chomnunti P, Dissanayake A, Drenkhan R, Friberg H, Frøslev TG, Halwachs B, Hartmann M, Henricot B, Jayawardena R, Jumpponen A, Kauserud H, Koskela S, Kulik T, Liimatainen K, Lindahl BD, Lindner D, Liu J-K, Maharachchikumbura S, Manamgoda D, Martinsson S, Neves MA, Niskanen T, Nylinder S, Pereira OL, Pinho DB, Porter TM, Queloz V, Riit T, Sánchez-García M, Sousa Fde, Stefańczyk E, Tadych M, Takamatsu S, Tian Q, Udayanga D, Unterseher M, Wang Z, Wikee S, Yan J, Larsson E, Larsson K-H, Kõljalg U, Abarenkov K (2014). Improving ITS sequence data for identification of plant pathogenic fungi. Fungal Diversity.

[ref-38] Ozer G, Bayraktar H (2018). Genetic diversity of Fusarium oxysporum f. sp cumini isolates analyzed by vegetative compatibility, sequences analysis of the rDNA IGS region and iPBS retrotransposon markers. Journal of Plant Pathology.

[ref-39] Özer G, Bayraktar H, Baloch FS (2016). iPBS retrotransposons ‘A Universal Retrotransposons’ now in molecular phylogeny of fungal pathogens. Biochemical Systematics and Ecology.

[ref-40] Patriarca A (2016). Alternaria in food products. Current Opinion in Food Science.

[ref-41] Peakall R, Smouse PE (2012). GenAlEx 6.5: genetic analysis in Excel. Population genetic software for teaching and research–an update. Bioinformatics.

[ref-42] Pinto V, Patriarca A (2017). Alternaria species and their associated mycotoxins. Mycotoxigenic Fungi.

[ref-43] Raffaele S, Kamoun S (2012). Genome evolution in filamentous plant pathogens: why bigger can be better. Nature Reviews Microbiology.

[ref-44] Ramallo E, Kalendar R, Schulman AH (2008). Reme1, a Copia retrotransposon in melon, is transcriptionally induced by UV light. Plant Molecular Biology.

[ref-45] Sanchez DH, Gaubert H, Drost HG, Zabet NR, Paszkowski J (2017). High-frequency recombination between members of an LTR retrotransposon family during transposition bursts. Nature Communications.

[ref-46] Schulman AH, Kalendar R (2005). A movable feast: diverse retrotransposons and their contribution to barley genome dynamics. Cytogenetic and Genome Research.

[ref-47] Shamim M, Kumar P, Kumar RR, Kumar M, Kumar RR, Singh KN (2017). Assessing fungal biodiversity using molecular markers. Molecular markers in mycology.

[ref-16] Sivolap IuM, Kalendar RN, Chebotar SV (1994). The genetic polymorphism of cereals demonstrated by PCR with random primers. Cytology and Genetics.

[ref-55] Šķipars V, Siaredzich M, Belevich V, Bruņeviča N, Brũna L, Ruņgi̧s DE (2018). Genetic differentiation of Phoma sp. isolates using retrotransposon-based iPBS assays. Environmental and Experimental Biology.

[ref-48] Somma S, Amatulli MT, Masiello M, Moretti A, Logrieco AF (2019). Alternaria species associated to wheat black point identified through a multilocus sequence approach. International Journal of Food Microbiology.

[ref-49] Teo CH, Tan SH, Ho CL, Faridah QZ, Othman YR, Heslop-Harrison JS, Kalendar R, Schulman AH (2005). Genome constitution and classification using retrotransposon-based markers in the orphan crop banana. Journal of Plant Biology.

[ref-50] Tralamazza SM, Piacentini KC, Iwase CHT, Rocha LdO (2018). Toxigenic Alternaria species: impact in cereals worldwide. Current Opinion in Food Science.

[ref-51] Underwood CJ, Henderson IR, Martienssen RA (2017). Genetic and epigenetic variation of transposable elements in Arabidopsis. Current Opinion in Plant Biology.

[ref-52] Vicient C, Jaaskelainen M, Kalendar R, Schulman A (2001). Active retrotransposons are a common feature of grass genomes. Plant Physiology.

[ref-53] Vicient C, Kalendar R, Schulman A (2005). Variability, recombination, and mosaic evolution of the barley BARE-1 retrotransposon. Journal of Molecular Evolution.

[ref-54] Vos P, Hogers R, Bleeker M, Reijans M, Van de Lee T, Hornes M, Frijters A, Pot J, Peleman J, Kuiper M (1995). AFLP: a new technique for DNA fingerprinting. Nucleic Acids Research.

[ref-56] Vukich M, Schulman AH, Giordani T, Natali L, Kalendar R, Cavallini A (2009). Genetic variability in sunflower (Helianthus annuus L.) and in the Helianthus genus as assessed by retrotransposon-based molecular markers. Theoretical and Applied Genetics.

[ref-57] Vuorinen A, Kalendar R, Fahima T, Korpelainen H, Nevo E, Schulman A (2018). Retrotransposon-based genetic diversity assessment in wild Emmer wheat (Triticum turgidum ssp. dicoccoides). Agronomy.

[ref-58] Waugh R, McLean K, Flavell AJ, Pearce SR, Kumar A, Thomas BB, Powell W (1997). Genetic distribution of Bare-1-like retrotransposable elements in the barley genome revealed by sequence-specific amplification polymorphisms (S-SAP). Molecular and General Genetics.

[ref-59] Wenderoth M, Garganese F, Schmidt-Heydt M, Soukup ST, Ippolito A, Sanzani SM, Fischer R (2019). Alternariol as virulence and colonization factor of Alternaria alternata during plant infection. Molecular Microbiology.

[ref-60] Williams JGK, Kubelik AR, Livak KJ, Rafalski JA, Tingey SV (1990). DNA polymorphisms amplified by arbitrary primers are useful as genetic markers. Nucleic Acids Research.

[ref-61] Woudenberg JH, Seidl MF, Groenewald JZ, De Vries M, Stielow JB, Thomma BP, Crous PW (2015). Alternaria section Alternaria: species, formae speciales or pathotypes?. Studies in Mycology.

[ref-62] Wu J, Xie XW, Shi YX, Chai A, Wang Q, Li BJ (2019). Analysis of pathogenic and genetic variability of Corynespora cassiicola based on iPBS retrotransposons. Canadian Journal of Plant Pathology.

[ref-63] Xu J (2016). Fungal DNA barcoding. Genome.

[ref-64] Zietkiewicz E, Rafalski A, Labuda D (1994). Genome fingerprinting by simple sequence repeat (SSR)-anchored polymerase chain reaction amplification. Genomics.

